# Effects of Chlorhexidine-Encapsulated Mesoporous Silica Nanoparticles on the Anti-Biofilm and Mechanical Properties of Glass Ionomer Cement

**DOI:** 10.3390/molecules22071225

**Published:** 2017-07-21

**Authors:** Huiyi Yan, Hongye Yang, Kang Li, Jian Yu, Cui Huang

**Affiliations:** The State Key Laboratory Breeding Base of Basic Science of Stomatology (Hubei-MOST) & Key Laboratory for Oral Biomedical Ministry of Education, School & Hospital of Stomatology, Wuhan University, Wuhan 430079, China; huiyiyan@whu.edu.cn (Hu.Y.); yanghongye@whu.edu.cn (Ho.Y.); michaelli@whu.edu.cn (K.L.); yujiandoctor@whu.edu.cn (J.Y.)

**Keywords:** chlorhexidine, mesoporous silica nanoparticles, glass ionomer cement, biofilm, *Streptococcus mutans*

## Abstract

One of the primary causes for the failure of glass ionomer cement (GIC) is secondary caries. To enhance the anti-microbial performance of GIC without affecting its mechanical properties, chlorhexidine (CHX) was encapsulated in expanded-pore mesoporous silica nanoparticles (pMSN) to synthesize CHX@pMSN. CHX@pMSN was added at three mass fractions (1%, 5%, and 10% (*w/w*)) to GIC powder as the experimental groups. Pure GIC was set as the control group. The mechanical and anti-biofilm properties of GIC from each group were tested. The results demonstrated that CHX was successfully encapsulated on/into pMSN, and the encapsulating efficiency of CHX was 44.62% in CHX@pMSN. The anti-biofilm ability was significantly enhanced in all experimental groups (*p* < 0.001) compared with that in the control group. CHX was continuously released, and anti-biofilm ability was maintained up to 30 days. In addition, the mechanical properties (compressive strength, surface hardness, elastic modulus, water sorption, and solubility) of 1% (*w/w*) group were maintained compared with those in the control group (*p* > 0.05). In conclusion, adding 1% (*w/w*) CHX@pMSN to GIC led to conspicuous anti-biofilm ability and had no adverse effect on the mechanical properties of this restorative material. This study proposes a new strategy for preventing secondary caries by using CHX@pMSN-modified GIC.

## 1. Introduction

Glass ionomer cement (GIC) was first introduced by Wilson and Kent [1] in 1970s, and since then, it has been widely used in esthetic dentistry and has attracted increasing research attention. GIC can bind directly to the tooth structure via interaction with the natural apatite without light curing or rotary instruments [2]; as such, GIC is convenient and popular in less-developed regions and for patients with dental phobia. Moreover, GIC continuously releases fluoride, which remineralizes dentin and enamel surrounding the restorative materials and suppresses bacterial activities [3]. Hence, GIC is preferred over other tooth-color materials especially when used for senile caries, childhood caries and atraumatic restorative treatment [4].

Dental caries is principally derived from cariogenic bacteria (especially *Streptococcus mutans*) which participate in biofilm formation and subsequent cariogenesis [5]. *Streptococcus mutans* (*S. mutans*) and other cariogenic bacteria could invade the GIC-dentin interfaces via microleakage to induce the occurrence of secondary caries, which will eventually result in the failure and replacement of GIC [6]. The unfavorable antibacterial ability restricted the application of traditional GIC [7,8]. Hence, the antimicrobial capability of GIC should be improved to combat dental caries and prolong the service life of this restorative material.

GIC has been modified using various antimicrobial agents, such as quaternary ammonium methacrylates, ethanolic extracts of propolis, epigallocatechin-3-gallate, and chlorhexidine (CHX), which can improve the antimicrobial property of conventional GIC to some extent [4,9,10,11,12,13]. CHX, a cationic bis-biguanide, is the “golden standard” when evaluating other antimicrobial agents [14,15]. CHX possesses wide-spectrum antibacterial activity to resist bacterial colonization [16] and nontoxicity toward mammalian cells [17]; thus, CHX is widely used in dentistry to prevent dental plaques and to control infection. Usually CHX is simply mixed with GIC to enhance the anti-bacterial property; however, the physical and mechanical properties of GIC are also changed in the process [4,13,18,19,20,21]. However, the effect is desired that long-term release of CHX in GIC may help preventing secondary caries and it would be beneficial for clinical promotion of the dental application of GIC. Therefore, an ingenious addition of CHX to GIC should be developed.

Mesoporous silica nanoparticles (MSN), labeled with stable framework, high specific surface area, tailored mesoporous structure, and favorable biocompatibility, are an ideal host for multifarious molecules [22]. MSN was first proposed by Yanagisawa et al. in 1990 [23], and has been widely used as a drug delivery system for antibiotics, anti-inflammatory agent, and anti-cancer drugs since these particles can function as reservoirs for sustained drug release [17,24,25,26]. Simply mixing additives would alter the mechanical properties of GIC [4,13,18,19,20,21]; as such, we speculate that the good dispersibility of MSN [27] might be favorable for encapsulating and continuously releasing CHX without reducing the mechanical performance of GIC. In recent years, MSN has also been applied in dental materials as drug carriers. For instance, Jung-Hwan Lee et al. loaded amphotericin B into the MSN-incorporated poly (methyl methacrylate) dental resin and observed a long-term antimicrobial effect for 2 weeks [28]. Moreover, scientists have developed MSN carrying CHX to fight against oral bacterial biofilms [15,29]. However, these studies only reported anti-biofilm property for 24 h, and to the best of our knowledge, no reports are available on GIC modification with CHX-containing-MSN.

This study aims to (1) establish a CHX delivery system based on expanded-pore mesoporous silica (CHX@pMSN); (2) develop a new strategy that endows GIC with anti-biofilm ability by appropriate addition of CHX@pMSN; and (3) evaluate the effects of CHX@pMSN on the mechanical properties of the modified GIC. The null hypothesis states that incorporating different amounts of CHX@pMSN has no significant influence on the anti-biofilm capability and mechanical performance of GIC.

## 2. Results and Discussion

### 2.1. Characterization of pMSN and CHX@pMSN

**Figure 1:**
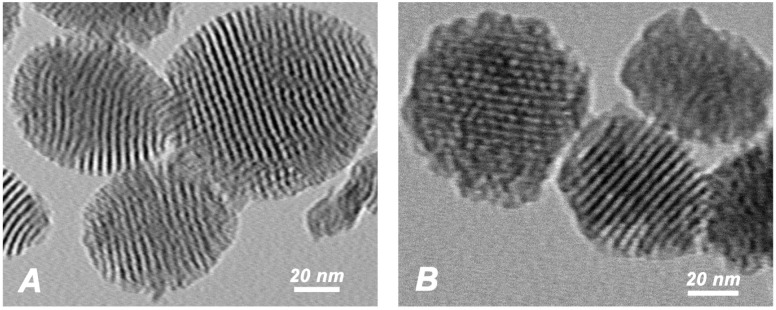
TEM images of (**A**) pMSN; and (**B**) CHX@pMSN.

In this study, pMSN and CHX@pMSN were fabricated. The transmission electron microscopy (TEM) image of pMSN particles in Figure 1A shows that pMSN particles are typical spherical or elliptical ball-shaped particles with diameters between 50 nm and 100 nm, showing ordered nanopores and channel framework. Figure 1B shows that the structure of the margin and inner mesoporosity for CHX@pMSN were obscured and less clear than pure pMSN particles, mainly on account of the adsorption of CHX on the surface of nanoparticles and permeation into pMSN cavities. It also shows good dispersibility of the nanoparticles before incorporating in GIC matrix.

**Figure 2:**
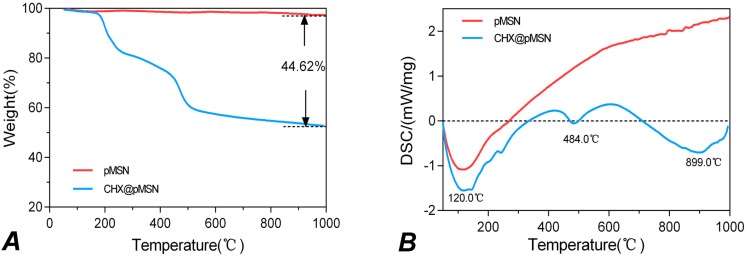
The thermogravimetric analysis for pMSN and CHX@pMSN shows (**A**) TGA curves; and (**B**) DSC curves.

The thermogravimetric analysis (TGA) curves and derivative differential scanning calorimetry (DSC) curves for pMSN and CHX@pMSN are shown in Figure 2. For pMSN, a downward crest is found at 120 °C in the DSC curve (Figure 2B), indicating the evacuation of physically adsorbed water under 120 °C and the desorption of chemically bonded water above 120 °C [17,30]. These two parts of water jointly contribute to the weight loss (2.67%) of blank pMSN particles (Figure 2A). For CHX@pMSN, besides the similar weight loss at approximately 120 °C, another obvious downward crest in the DSC curve could be observed at 484 °C (Figure 2B), which might refer to the decomposition of organic matter, indicating the desorption of CHX. Accordingly, the loading amount was calculated as 44.62% to the total weight of CHX@pMSN particles (Figure 2A). Michał Moritz and co-workers reported the loading capacity ranging from 8.55% to 41.67% of five different mesoporous materials as the carrier for chlorhexidine [17]. Our result showed that CHX was successfully encapsulated on/into pMSN, and a relatively high loading amount of CHX (44.62%) was achieved. This could be due to that MSN with expanded-pore and good dispersibility was synthesized in the present study. These characteristics might be favorable for encapsulating more CHX to prevent secondary caries.

### 2.2. Anti-Biofilm Property

#### 2.2.1. MTT Assay and FESEM Observation

**Figure 3:**
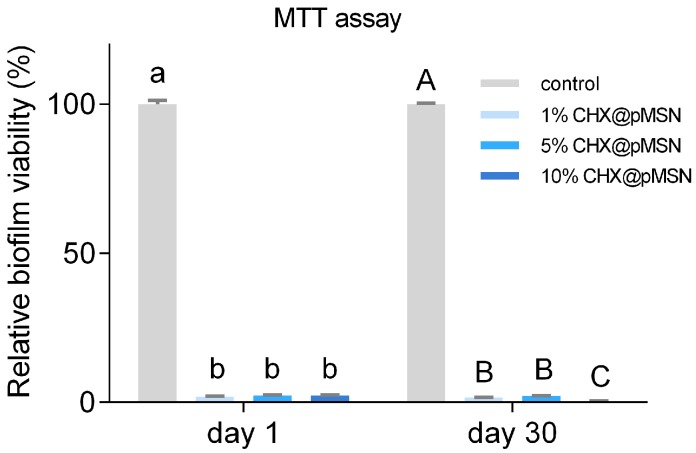
Relative biofilm viability of control, 1%, 5%, and 10% CHX@pMSN groups by MTT assay on (**A**) day 1; and (**B**) day 30. Data are shown as mean ± standard deviation. The groups labeled with the same letters have no significant difference (*p* > 0.05).

**Figure 4:**
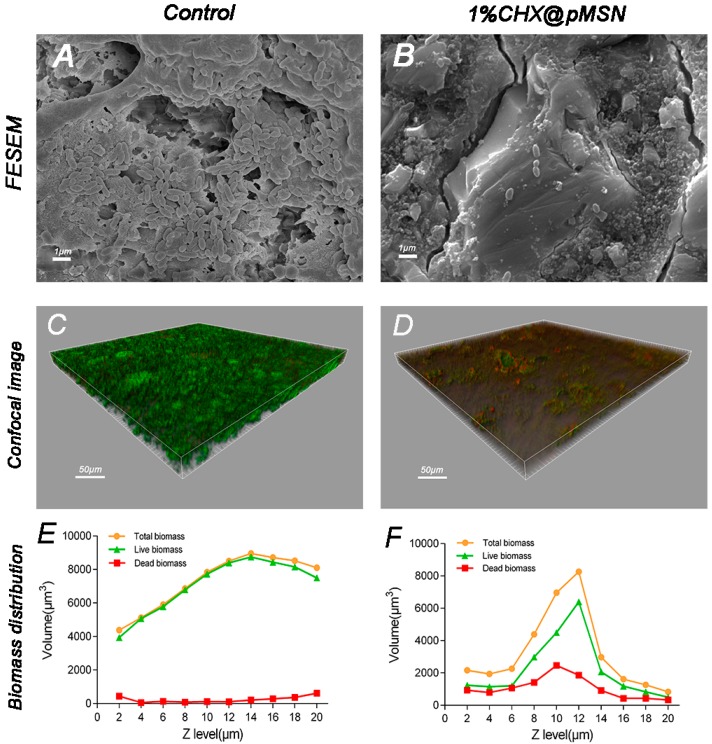
FESEM and CLSM evaluation of *S. mutans* biofilms. (Top) FESEM images of (**A**) control; and (**B**) 1% CHX@pMSN groups. (Middle) CLSM 3D overlay projections of (**C**) control; and (**D**) 1% CHX@pMSN groups. (Bottom) Corresponding distribution of live/dead bacteria biomass at each layer of Z-stack from (**E**) control; and (**F**) 1% CHX@pMSN groups.

T3-(4,5-Dimethylthiazol-2-yl)-2,5-diphenyltetrazolium bromide (MTT) assay method was adopted to demonstrate the inhibition of biofilm on the surface of GIC modified by CHX@pMSN, and the result is shown in Figure 3. Each experimental group exhibited identical outstanding ability to inhibit biofilm formation relative to the control group (*p* < 0.001), independent of the weight percent of CHX@pMSN. The mean reduction of relative biofilm viability for three experimental groups were 97.81% and 98.56% on day-1 and day-30, respectively. Figure 4A,B showed typical field emission scanning electron microscopy (FESEM) images of the *S. mutans* biofilm-coated specimens of control and 1% CHX@pMSN group. The produced biofilm almost covered the entire surface of specimen from the control group, while the specimens from 1% CHX@pMSN group were attached with a few scattered bacteria which might not be defined as “biofilm”. This phenomenon showed that the released amount of CHX from GIC containing 1% (*w/w*) CHX@pMSN was strong enough to inhibit the growth of *S. mutans*, thus the formation of biofilm was interrupted.

#### 2.2.2. CLSM Observation

Confocal laser scanning microscopy (CLSM) was used to analyze the stained live/dead bacterial viability of specimens from each group. Figure 4C,D show illustrative 3D overlay images of the control and 1% CHX@pMSN groups. Many live *S. mutans* adhered to the surface of control group specimens, colonized, and formed biofilms. Given that GIC has intrinsic F^−^ release property, which can inhibit the metabolic process of bacteria [3], a few dead bacteria (red fluorescence) could be found on the control group (Figure 4C). However, for 1% CHX@pMSN group, not only live bacteria but also the total volume of germs was significantly less than the control ones, which demonstrated the same tendency as FESEM images. In addition, Figure 4E,F show the distribution of live/dead bacteria biomass at each layer of Z-stack from Figure 4C (control) and Figure 4D (1% CHX@pMSN) respectively. As total biomass of every layer and the total biofilm biomass (area under the curve) from 1% CHX@pMSN group were significantly less than that in the control group, the line chart shows that *S. mutans* tended not to adhere to CHX@pMSN-modified GIC surface. Furthermore, both the absolute amount and proportion to total biomass of dead bacteria in 1% CHX@pMSN group were greater than those in the control group, indicating that the CHX@pMSN-modified GIC also inactivated bacteria colonized on the surface. On the other hand, the biomass at 20 μm of Z stack in the control group was still very high whereas little bacteria were observed in the 1% CHX@pMSN group at the same layer. This implies that the biofilm of the control group was far beyond 20 μm and much thicker than that in the 1% CHX@pMSN group. From these results, more germs might have been killed by CHX before attaching to the surface of experimental specimens.

MSN encapsulating CHX was added in dental composites in previous study [15], it only demonstrated the antibacterial ability in 24 h. Another study loaded amphotericin B into the MSN-incorporated poly (methyl methacrylate) dental resin, and a long-term antimicrobial effect was observed for 2 weeks [28]. However, the eminent antibacterial ability of experimental groups in the present study could be maintained up to 30 days (Figure 3). This may result from the continuous release of CHX by pMSN, owing to the mesoporous structure. Consequently, CHX@pMSN has successfully been introduced into GIC to suppress *S. mutans* biofilm and thereby inhibits the development of secondary caries. Moreover, the in vivo anti-biofilm ability of the CHX@pMSN-modified GIC in a longer time would be tested in the follow-up study.

### 2.3. Mechanical and Physical Properties

#### 2.3.1. Compressive Strength, Surface Vickers Hardness, and Elastic Modulus

Many researches have shown that the mechanical behavior of GIC would be definitely changed by additives, such as CHX and nanoparticles [19,31,32,33,34]. As a restorative material available in various conditions and usually applied as fissure sealing for posterior tooth [8], modified-GIC ought to have adequate mechanical and physical properties to resist occlusal forces while enhancing antibacterial ability. Josh Slane et al. reported that when incorporated into acrylic bone cement, MSN tended to increase the flexural modulus and compressive strength but decrease the flexural strength and fracture toughness [35]. It has shown bidirectional effect of MSN on biomaterials. Therefore, the influence of CHX@pMSN addition on mechanical performance of GIC should be measured in our study.

**Figure 5:**
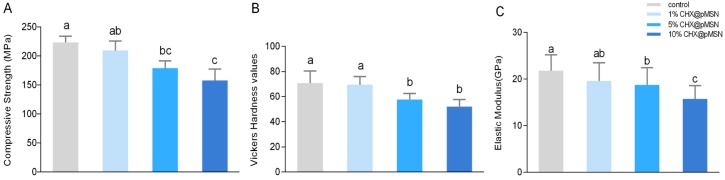
Mechanical properties of control, 1%, 5%, and 10% CHX@pMSN groups. (**A**) Compressive strength; (**B**) Vickers hardness; and (**C**) elastic modulus. Data are shown as mean ± standard deviation. The groups labeled with the same letters have no significant difference (*p* > 0.05).

The compressive strength (Figure 5A), surface Vickers hardness (Figure 5B), and elastic modulus (Figure 5C) of each group are shown in Figure 5. As shown in the figure, 5% and 10% (*w/w*) addition of CHX@pMSN into the powder of GIC significantly decreased the three mentioned performance indicators. However, addition up to 1% (*w/w*) retained the compressive strength, surface Vickers hardness, and elastic modulus of the GIC. The result implies that 1% (*w/w*) CHX@pMSN-modified GIC could provide a significant anti-biofilm performance without affecting the mechanical properties of GIC, suggesting a considerable potential for clinical use. In a previous study, CHX@MSN with the same CHX content endowed dental composites with significantly higher flexural strength than those directly mixed with CHX [15]. In this way, MSN does have the ability to enhance the properties of matrix. Whether CHX@pMSN will endow GIC with favorable anti-biofilm ability and unaffected or improved mechanical performance when being added in lower concentration (e.g., 0.1% and 0.5% (*w/w*)) will be tested in our follow-up study. The mechanical performance of GIC decreased when the incorporation amount of CHX@pMSN exceeded 5%. Elzbieta Horszczaruk et al. reported that large agglomerates of nanoparticles may become weak zones in cement matrix [36]. The high concentration of CHX@pMSN may also form agglomerates in the present study. The factors behind this phenomenon should further be explored.

#### 2.3.2. FESEM Observation of Fracture Surfaces

**Figure 6:**
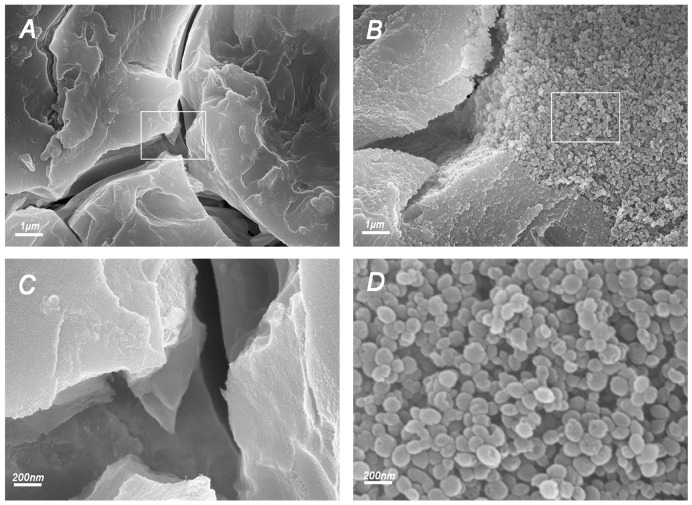
FESEM images of fracture surfaces from (**A**) control; and (**B**) 10% CHX@pMSN groups. (**C**,**D**) are magnified pictures of rectangles in (**A**,**B**) respectively.

**Figure A1:**
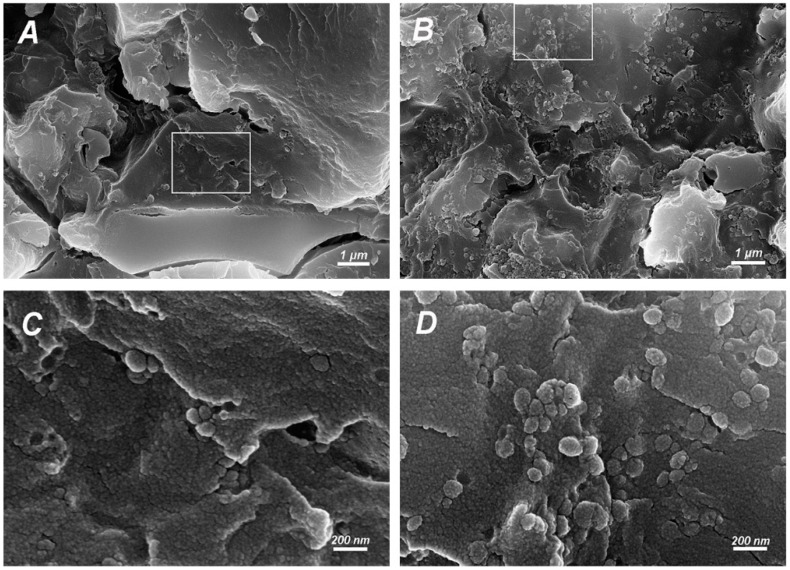
FESEM images of fracture surfaces from (**A**) 1% CHX@pMSN; and (**B**) 5% CHX@pMSN groups. (**C**,**D**) are magnified pictures of rectangles in (**A**,**B**) respectively. The nanoparticles dispersed uniformly in the GIC matrix and formed small agglomeration in 1% CHX@pMSN group. The agglomeration became larger in 5% CHX@pMSN group, however the dispersion of nanoparticles was uniform.

FESEM demonstrated the microstructure of the fracture surfaces of specimens from each group. Images from the control and 10% CHX@pMSN groups have shown significant difference (Figure 6), which might explain the phenomenon of decreased mechanical properties in high-addition concentration groups. Images from the 1% and 5% CHX@pMSN groups are shown in Figure A1. Comparing with the control group (Figure 6A,C), the researchers observed the formation of agglomerates, besides the relative homogeneous matrix, in 10% CHX@pMSN group (Figure 6B,D). Figure 6D shows more details with an enlarged scale for Figure 6B. The diameters of nanoparticles ranged from 50 nm to 100 nm, and the shapes were round or oval, matching the morphological characteristics of CHX@pMSN (as shown in Figure 1). However, for the control group, the cement was still homogeneous, even in the magnified picture (Figure 6C). This finding shows that the high incorporation amount of CHX@pMSN tended to aggregate together when mixing into basic aluminum fluoride silicate glass powder or blending with polyacrylic acid liquid. Moreover, the interface between agglomerates and matrix (GIC) might act as weak area and adversely affect the physical strength. The relative amount of Al^3+^ in the mixture decreases with increasing concentration of CHX@pMSN nanoparticles without Al^3+^. Adding excessive CHX@pMSN might affect the inner properties of GIC because Al^3+^ can improve the material strength by forming three-dimensional crosslinks with polyacrylic acid and other released ions [37,38]. In addition, the matrix GIC seemed to be insufficient to embed every nanoparticle, and the presence of high concentration of CHX@pMSN might act as a macroscopic barrier, blocking the complete contact of powder and liquid of GIC. Moreover, the synthetic MSN may not have functional groups to interact with the matrix. As a result, non-uniform component structure and uneven stress distribution finally formed. For all the reasons above, the amount of CHX@pMSN mixed into GIC should not be too much. In our future work, lower concentration of CHX@pMSN could be adopted to obtain adequate dispersion and MSN could be modified with functional groups to increase the chemical interaction with matrix.

#### 2.3.3. Water Sorption and Solubility

Given that oral cavity is a moisture condition, the network of restorative materials may absorb water and chemicals from the environment and may release components to its surroundings in turn [39]. The fact that GIC is hydrophilic and sensitive to moisture may give rise to water sorption or even hydrolytic degradation, resulting in reduced mechanical performance and shortened service life in the end [40,41]. Therefore, whether addition of CHX@pMSN would bring adverse effects to water sorption and solubility of GIC should be evaluated.

**Figure 7:**
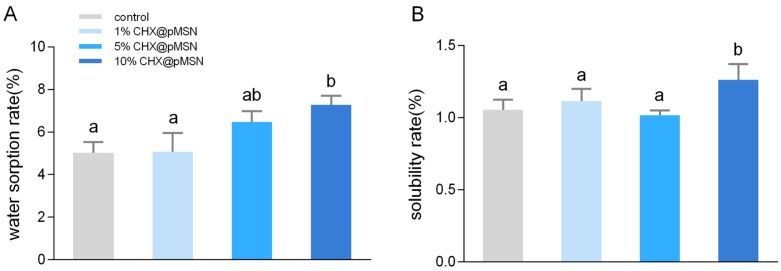
Water sorption rate (**A**) and solubility rate (**B**) of control, 1%, 5%, and 10% CHX@pMSN groups. Data are shown as mean ± standard deviation. The groups labeled with the same letters have no significant difference (*p* > 0.05).

Water sorption rate and solubility for 7 days of each group are plotted in Figure 7. With increasing added amount of CHX@pMSN, the water sorption rate also increased, but no statistical difference among the control, 1%, and 5% CHX@pMSN groups (*p* > 0.05) was found (Figure 7A). These three groups also showed no significant difference on solubility rate (*p* > 0.05) (Figure 7B), while both indicators in 10% CHX@pMSN increased (*p* < 0.05). Adding CHX@pMSN exceeding a certain concentration might increase the water sorption rate because CHX@pMSN possesses a nanoporous structure, which provides several pathways for water to infiltrate into GIC. Moreover, high concentration of CHX@pMSN might block the powder–liquid mixing process, thereby promoting the dissolution of the unreacted ingredient.

#### 2.3.4. Release Profile of CHX@pMSN-Modified GIC

**Figure 8:**
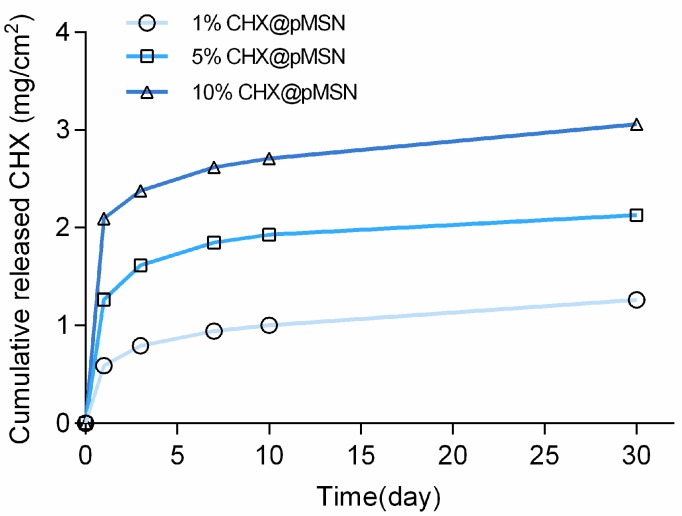
Release profile of cumulative CHX of 1%, 5%, and 10% CHX@pMSN groups at time points of 1, 3, 7, 10, and 30 days. All specimens were stored in distilled water at 37 °C.

The cumulative released CHX quantity of each experimental group from 1 day to 30 days is shown in Figure 8. A rapid release at the first day was observed, then the release speed decreased and was kept at a relatively stable level until 30 days for all three groups. Even so, the released CHX from 1% CHX@pMSN-modified GIC was still strong enough to inhibit *S. mutans* biofilm formation after 30 days, just as Figure 3 shows. Thus, CHX would be continuously released from the experimental specimens over time, and the anti-biofilm ability could be preserved.

An interesting phenomenon showed that the cumulative released amount of CHX in 5% and 10% CHX@pMSN group were significantly more but not five or ten times higher than that in 1% CHX@pMSN group. This phenomenon could be attributed to that most of the CHX@pMSN nanoparticles were embedded in the cement, the amount of CHX@pMSN on the GIC surface which could directly contact with water to release CHX was limited. For the same reason, 30 days’ continuous releasing behavior of CHX from modified GIC was achieved, just as Figure 8 showed. In addition, as it is shown in Figure A1, the nanoparticles in 1% CHX@pMSN-modified GIC demonstrated the uniform dispersion in the cement matrix, and it would increase the relative effective surface to release CHX. A longer releasing performance of CHX will be evaluated in our future study to verify our strategy.

## 3. Materials and Methods

### 3.1. Materials

CHX diacetate salt hydrate, tetraethyl orthosilicate (TEOS), hexadecyl trimethyl ammonium bromide (CTAB), mesitylene (TMB), and MTT were purchased from Sigma-Aldrich (St. Louis, MO, USA). A powder–liquid version of a conventional glass ionomer cement (GIC; Fuji IX, GC Corp., Tokyo, Japan) served as the parent material. All chemicals were used as received without further purification.

### 3.2. Preparation and Characterization of CHX@pMSN

pMSN was synthesized based on a previously reported technique [42] with a slight modification. Briefly, 7 mL of TMB and 1 g of CTAB were dissolved in a solution containing 480 mL of water and 3.5 mL of 2 mol/L NaOH. The mixture was stirred at 80 °C for 4 h. A 5 mL volume of TEOS was added to the solution, and the mixture was stirred vigorously for another 2 h. The synthetic product was a white precipitate, and it was centrifuged and washed with deionized water and ethanol three times each and oven-dried at 60 °C overnight. The white product was calcined in air at 550 °C for 5 h to remove CTAB and TMB.

The encapsulation process was performed as follows. CHX/ethanol solution (10 mg/mL) was mixed with 50 mg of pMSN, sonicated for 30 min and gently shaken for 24 h. The mixture (CHX@pMSN) was centrifuged, vacuum-dried, and stored under room temperature until use.

The ultrastructural characteristics of pMSN and CHX@pMSN were examined using TEM (JEM-1230; JEOL, Tokyo, Japan). The average weight percentage of CHX on pMSN was analyzed through a thermogravimetric analyzer (STA449F3, NETZSCH, Selb, Germany) by measuring the weight loss from room temperature to 1000 °C at a heating rate of 10 °C /min.

### 3.3. Preparation of Experimental GIC

CHX@pMSN was added into GIC powder at 1%, 5%, and 10% (*w/w*) as the experimental groups, set as 1%CHX@pMSN, 5%CHX@pMSN, and 10%CHX@pMSN group, respectively. Pure GIC was set as control group. According to the manufacturer’s instructions, the cement was produced by spatulation of the powder mixture into the liquid at recommended powder/liquid ratio of 3.6 g/1.0 g. One drop of liquid was mixed with one pre-measured spoon of powder within 30 s. Then the mixed material was put into molds within 2 min. The polytetrafluoroethylene mold with circular holes (Ø 8 mm, H 2 mm) was used as a model for specimen preparation. The chemical-cured specimens were sequentially wet-polished with 600-, 800-, 1000-, 1200-, 1500-grit carborundum papers to produce slabs 1-mm thick. After sonication, washing and disinfection under ultraviolet light, the slabs were stored in sterile glass bottles for 24 h before use. A total of 104 disk specimens were produced (*n* = 26 each group).

### 3.4. Specimen Preparation for Antibiofilm Test

Fifteen specimens from each group were used to cultivate biofilm in vitro, nine for MTT assay, three for CLSM analysis, and three for FESEM analysis. *S. mutans* UA159, provided by the School of Stomatology of Wuhan University, was anaerobically incubated overnight at 37 °C for 24 h in a brain–heart infusion (BHI) broth (BD, Sparks, MD, USA). The disks were transferred into the wells of a 24-well plate, then 10 μL of *S. mutans* cell suspension (adjusted as 10^8^ CFU/mL in advance) and 1 mL of BHI with 1% sucrose were added to each well. After anaerobic incubation at 37 °C for 24 h, biofilm-coated specimens were obtained, and the loose-adherent bacteria on the surface of specimens were gently washed away with sterile phosphate buffered saline (PBS) (pH 7.3).

#### 3.4.1. MTT Assay of CHX@pMSN-Modified GIC at 1 Day and 30 Days

For the 1-day test, the biofilm-coated specimens from each group were transferred to a 12-well plate containing 1 mL of MTT solution (0.5 mg/mL). After anaerobic incubation at 37 °C for 1 h, the MTT solution was replaced with 1 mL dimethyl sulfoxide (DMSO). The intracellular insoluble purple formazan induced by MTT could be dissolved by DMSO. The plate was gently shaken for 10 min, and the OD570 value of the supernatant was measured using a spectrophotometer (Powerwave 340, Bio-tek Instruments, Winooski, VT, USA).

After measurement, the same specimens were transferred into 5 mL of PBS in a plastic centrifuge tube, sonicated, washed, and disinfected under ultraviolet light again. Then, the disinfected specimens were placed in a 24-well plate and submerged in 1 mL of daily replaced artificial saliva for 30 days, and then the MTT assay was tested again. This experiment was repeated three times (*n* = 9).

#### 3.4.2. Confocal Laser Scanning Microscopy Analysis

Three biofilm-coated specimens from each group were stained by the live/dead bacterial viability kit (Molecular Probes, Invitrogen, Carlsbad, CA, USA) for 15 min. After gently rinsing with PBS, *S. mutans* adhered on the specimen were analyzed by a CLSM (Fluoview FV1200, Olympus, Tokyo, Japan) at 40× magnification. The excitation at 488 nm wavelengths emitted green fluorescence of live bacteria stained by SYTO-9, while the excitation at 543-nm wavelengths emitted red fluorescence of dead bacteria stained by propidium iodide. A continuous scanning along the Z-stack produced 10 CLSM images from the bottom to the top of the biofilm (20 μm in total). 3D overlay images were reconstituted by Imaris 7.2.3 software (Bitplane, Zürich, Switzerland), while the distributions of live and dead bacteria at each layer were also plotted respectively.

#### 3.4.3. FESEM Analysis

Three biofilm-coated disks from each group were prepared for FESEM analysis. The specimens were fixed in 2.5% glutaraldehyde (0.1 mol/L cacodylate buffer, pH 7.2) for 4 h at 4 °C. Specimens were dehydrated in a series of ethanol washes (30%, 50%, 70%, 80%, 90% for 20 min respectively, 100% for 20 min twice), and air dried in a desiccator. Then, they were mounted on copper stabs and coated with gold for 2 min. The biofilms formatted on the materials were visualized under FESEM (Sigma, Zeiss, Germany). The fracture surface of each group was also observed respectively under FESEM after being mounted on the copper stabs and sputtered with gold.

### 3.5. Surface Microhardness and Nanoindentation

Five disks from each group (control, 1%, 5%, and 10% CHX@pMSN) were subjected to a digital microhardness tester (HXD-100TMC/LCD, Taiming Inc., Shanghai, China) under a load of 50 g for 10 s. Five spots were randomly selected and tested in each specimen.

Two specimens for each group were subjected to a nanoindentation test (TI 950 TriboIndenter, Hysitron, Inc., Eden Prairie, MN, USA). Ten spots were randomly selected and tested in each specimen. Elasticity modulus data were analyzed.

### 3.6. Compressive Strength

Additional five cylinder-shaped specimens (Ø 4 mm, H 6 mm) were prepared for each group, using a split standardized stainless steel mold. According to ISO standards [43], after the complete solidification of the cement for 1 h, the specimens were removed from the stainless-steel molds and wet-polished with 500-grit carborundum papers to obtain non-uniform ends. Then, the cylinders were stored in deionized water at 37 °C until 24 h after the mixing was completed before measuring the compressive strength using a universal testing machine (LRX plus, Lloyd Instruments, Bognor Regis, UK), with the crosshead speed set at 1.0 mm/min. The compressive strength (CS) (MPa) was calculated using the following equation:

CS = 4*F*/π*d*^2^ (1)

where *F* is the force (N) at fracture, *d* is the diameter of the specimen (mm).

### 3.7. Water Sorption and Solubility Rate

Four disk specimens from each group were dried in a desiccator until a stable weight (M1) (g) was obtained. After storage in distilled water for 7 days, the specimen was blot dried with filter paper and weighed (M2) (g) within 30 s to eliminate the influence of desiccation. The specimen was stored in a desiccator until a stable weight, noted as (M3), and its mass (g) was determined.

Water absorption and solubility rates were calculated using the following equations:

Water absorption rate = (M2 − M1)/M1 × 100%(2)

Solubility rate = (M1 − M3)/M1 × 100%(3)

### 3.8. CHX Release

Three disk specimens from each group were used to measure the release amount of CHX. The diameter and thickness of each specimen were recorded. Surface area was then calculated. Each dried specimen was immersed in 5 mL of sterile deionized water at 37 °C for 7 days. At time points of 1, 3, 7, 10, and 30 days, 1 mL of the solution was removed and replaced with distilled water. The removed aliquots were analyzed for the release of CHX by high-performance liquid chromatography (Agilent 1100, Palo Alto, CA, USA). The cumulative release of CHX from each specimen at each time point was calculated.

### 3.9. Statistical Analysis

Data derived from compressive strength test, surface microhardness test, nanoindentation, water absorption rate, solubility rate, and MTT assay at different time points were analyzed by one-way analysis of variance, and post-hoc multiple comparisons were conducted by Tukey’s test. All statistical analyses were performed using SPSS 20.0 (IBM SPSS Statistics 20, Armonk, NY, USA). The significance level was set at 0.05 for all tests.

## 4. Conclusions

In the present study, we synthesized pMSN to encapsulate CHX, a classic antimicrobial agent, and applied the CHX@pMSN prepared to modify dental conventional GIC for the first time. The results demonstrated that the CHX@pMSN-modified GIC at 1% (*w/w*) could perform sustained release of CHX and effectively inhibit the formation of *S. mutans* biofilm without affecting the mechanical properties of GIC. This study indicates that addition of 1% (*w/w*) CHX@pMSN into GIC is significantly potential as a new strategy against secondary caries, thus prolonging the service life of traditional GIC. The long-term impact of CHX@pMSN incorporation on GIC should be further evaluated in more complex scenarios through artificial aging methods (e.g., long-term storage, sodium hypochlorite treatment, and pH cycling). More desired incorporating strategies are still in need to endow GIC with strong antimicrobial ability and improved mechanical performance in our future research.
